# Anterior cruciate ligament reconstruction in a rabbit model using a silk-collagen scaffold modified by hydroxyapatite at both ends: a histological and biomechanical study

**DOI:** 10.1186/s13018-021-02281-0

**Published:** 2021-02-16

**Authors:** Fanggang Bi, Yangdi Chen, Junqi Liu, Yafei Wang, Danfeng Xu, Ke Tian

**Affiliations:** 1 Department of Orthopedic Surgery, The First Affiliated Hospital of Zhengzhou University, NO.1 Jianshe East Road, Zhengzhou, 450001 China; 2 Henan University of Chinese Medicine, NO.156 Jinshui East Road, Zhengzhou, 450001 China; 3 Department of Radiation Oncology, The First Affiliated Hospital of Zhengzhou University, NO.1 Jianshe East Road, Zhengzhou, 450001 China; 4 Department of Orthopedic Surgery, Shaoxing Central Hospital, NO.1 Huayu Road, Shaoxing, 312000 China

**Keywords:** Hydroxyapatite, Anterior cruciate ligament reconstruction, Tendon-bone healing, Osteointegration

## Abstract

**Background:**

To investigate osteointegration at the graft-bone interface and the prevention of osteoarthritis after anterior cruciate ligament (ACL) reconstruction using a silk-collagen scaffold with both ends modified by hydroxyapatite (HA) in a rabbit model.

**Methods:**

The HA/silk-collagen scaffold was fabricated using a degummed, knitted silk scaffold, collagen I matrix, and simulated body fluid (SBF). The HA/silk-collagen scaffold was rolled up to make a graft for replacing the native ACL in the experimental group (HA group), and the silk-collagen scaffold was used in the control (S group). All specimens were harvested at 16 weeks postoperatively to evaluate graft-bone healing and osteoarthritis prevention.

**Results:**

Histological staining revealed the massive formation of more mature bone at the tendon-bone interface, and immunohistochemistry staining revealed more collagen I and osteocalcin deposition in the HA group than in the S group. Higher signals indicating more bone mineral formation were detected in the HA group than in the S group, which was consistent with the results of biomechanical testing. Better osteoarthritis prevention was also observed in the HA group, indicating a more stable knee joint in the HA group than in the S group.

**Conclusion:**

The HA/silk-collagen scaffold promotes osteointegration at the tendon-bone interface after ACL reconstruction and has great potential for clinical applications.

**Supplementary Information:**

The online version contains supplementary material available at 10.1186/s13018-021-02281-0.

## Introduction

The anterior cruciate ligament (ACL) is an important factor in maintaining stability and enabling functional movements of the knee joint. Tear or rupture of the ACL is one of the most common injuries due to its biomechanical function during sports activities. There are 100,000-200,000 cases of ACL injury annually in the USA [1]. Since the ACL has a low capacity for regeneration, the current gold standard for treating ACL rupture is reconstruction with autografts [2]. Autografts, including hamstring grafts and bone-patellar tendon-bone grafts, have drawbacks, such as donor site morbidity, ligament laxity, and a high incidence of bone tunnel widening [3]. Alternatively, ACL reconstruction with allografts can avoid donor site morbidity for patients, but additional risks, including the risk of disease transmission, an immunogenic response, and a higher failure rate, should be considered [4]. Artificial synthetic grafts overcome some deficiencies of autografts and allografts and exhibit satisfactory results in the short term, but their long-term complications include graft rupture, chronic synovitis, foreign-body reactions, and poor tissue integration [5]. Therefore, research on ligament tissue engineering to develop an ideal biological scaffold for ACL reconstruction has become a focus in sports medicine.

An ideal biological scaffold for ACL reconstruction should be biocompatible and biodegradable, not only providing immediate joint stability but also promoting ligamentous tissue formation in the knee cavity and tendon-bone healing in the bone tunnel [2, 6]. Recently, silk-based scaffolds have been increasingly utilized for ligament regeneration because of their good biocompatibility, slow degradability, and remarkable mechanical strength [7]. The biocompatibility of silk can be promoted by degumming [8]. The combination of silk and a collagen sponge to mimic the native structure and composition of ligament extracellular matrix (ECM) exhibited satisfactory cellular infiltration and tissue regeneration. In a previous study, we fabricated a knitted silk-collagen scaffold and achieved promising results in a rabbit model of ACL reconstruction [2]. The knitted silk fibroin scaffold combined with the collagen matrix showed better neoligament regeneration and tendon-bone healing than autografts. However, the tendon-bone interface needs to be improved. Although the ingrowth of trabecular bone into the graft was observed at 16 weeks postoperatively [2], more tendon-bone healing is still required for osteointegration at the tendon-bone interface after ACL reconstruction.

Hydroxyapatite (HA) is generally known for its biocompatibility and has been proven to enhance tendon-bone healing in animal studies [9]. Methods for the surface modification of implants by HA include biomineralization, laser pulse deposition, plasma spraying, electrochemical deposition, electrophoretic deposition, and dip coating, among others [10–14]. Wang and colleagues demonstrated that the biocompatibility and osteointegration of a polyethylene terephthalate (PET) artificial ligament were significantly improved by coating the material with HA via the plasma-spraying technique, increasing the proliferation of cells and upregulating the expression of bone formation-related genes [12]. Li and colleagues modified the PET ligament by the dip-coating method and found that a commercial HA coating on the PET ligament had a positive effect on the induction of artificial ligament osteointegration in the bone tunnel [15]. However, commercial HA cannot replace natural bone mineralization because of its inability to resemble HA crystals in natural bone [16].

Therefore, the objective of the present study was to biomineralize HA on a silk-collagen scaffold and assesses the effect of HA on osteointegration at the graft-bone interface in an animal model of ACL reconstruction. Silk-collagen scaffolds with both ends modified by HA were used in a rabbit model of ACL reconstruction, and silk-collagen scaffolds were used as the control. The effects on osteointegration at the tendon-bone interface were verified by micro-CT, histology, and biomechanical testing. Meanwhile, the effects of the two scaffolds in terms of preventing osteoarthritis were also evaluated. The hypothesis is that the graft-bone healing process can be enhanced by HA biomineralization.

## Materials and methods

### Fabrication of silk-collagen scaffolds and HA/silk-collagen scaffolds

The fabrication of the silk-collagen scaffold has been previously described [2]. Briefly, raw silk fibers (*Bombyx mori*), provided by Zhejiang Cathaya International, Inc. (Hangzhou, China), were used to fabricate knitted scaffolds on a knitting machine. An aqueous solution containing 0.02 M Na_2_CO_3_ was used to remove sericin, the glue-like protein adhering to silk fibroin, by incubation at 90°C and 100°C for 60 min. The type I collagen solution was extracted and refined by neutral salt and dilute acid extractions from pig Achilles’ tendon. The silk fibroin scaffold was immersed in the collagen solution (15 mg/ml, 2 mm depth). It was frozen at −80°C and freeze-dried under vacuum for formation of the collagen sponge. Then, the scaffolds were crosslinked by dehydrothermal treatment [17]. Finally, the silk-collagen scaffolds were cut into rectangles 2×5 cm in size.

**Fig. 1:**
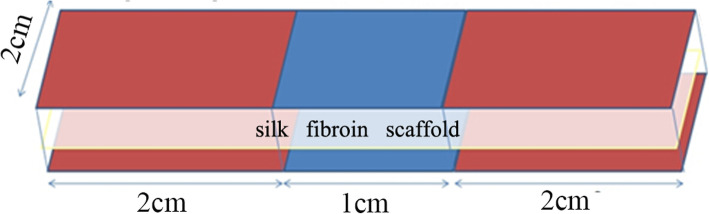
Schematic of the HA/silk-collagen scaffold with both ends modified by HA

Once the rectangular silk-collagen scaffolds (2×5 cm) were obtained, the distal and proximal 2-cm ends were immersed in simulated body fluid (SBF) for 4 days to nucleate bone-like nanostructured nonstoichiometric HA into self-assembling on the collagen fibers, as occurs in the biological process of neo-ossification (Fig. 1). Finally, all scaffolds were sterilized with γ irradiation.

### Experimental design

Twenty-four male New Zealand white rabbits weighing 2.5-3.0 kg (12 weeks old) were used in the present study. The Animal Care and Use Committee of Zhengzhou University approved the study protocol. All methods were performed in accordance with the relevant guidelines and regulations. The animals were randomly divided into two groups to undergo ACL reconstruction in the left hindlimb: animals in the S group (*n*=12) received silk-collagen scaffold, and those in the HA group (*n*=12) received HA/silk-collagen scaffolds. At 16 weeks postoperatively, all rabbits were sacrificed; half of the specimens in each group (*n*=6) were evaluated by micro-CT and biomechanical testing to determine osteointegration at the tendon-bone interface, and the other half (*n*=6) were evaluated by hematoxylin and eosin staining, safranin O staining, and immunohistochemical staining for collagen I, collagen III, and osteocalcin. All the cartilage surfaces of the femoral condyles were harvested for the assessment of osteoarthritis prevention.

### Surgical protocol

**Fig. 2:**
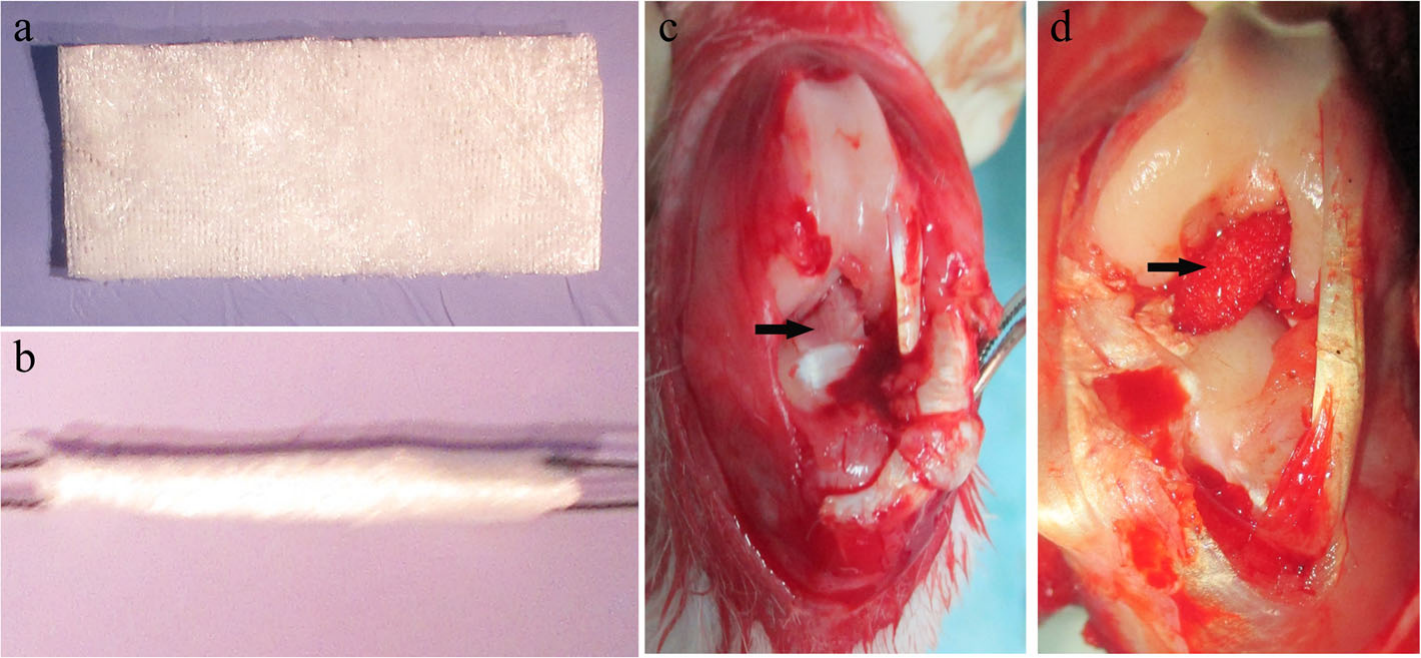
Gross view of the silk-collagen scaffold (**a**) and the scaffold rolled up for implantation (**b**). Gross view of the native ACL (**c**, the black arrow points to the native ACL) and the graft implanted into the knee joint to reconstruct the ACL (**d**, the black arrow points to the graft)

The surgical process of ACL reconstruction was performed under aseptic conditions. After general anesthesia was induced with an intravenous injection of pentobarbital sodium (30 mg/kg), the knee joint of the left hindlimb was exposed by lateral parapatellar arthrotomy. The native ACL was excised with a scalpel, and the tibial and femoral tunnels were created with a 2.0-mm drill at the ACL attachment site. The silk-collagen scaffold or HA/silk-collagen scaffold was carefully rolled up along the short axis to make a graft 2.0 mm in diameter and 50 mm in length. Then, the graft was passed through the bone tunnels, and both ends were fixed by sutures tied over screws in the tibia and femur. In the HA group, a 1-cm segment in the middle of the scaffold was ensured to be placed in the knee joint cavity. The incisions were sutured layer by layer, and all experimental animals were allowed to move freely in their cages postoperatively (Fig. 2a-d).

### Histological and immunohistological assessment of the tendon-bone interface

To evaluate osteointegration at the tendon-bone interface, the tibia and femur (*n*=6 for each group) were fixed in 10% paraformaldehyde immediately after collection. Then, the specimens were decalcified in 10% ethylenediaminetetraacetic acid (EDTA) for 4 weeks and embedded in paraffin. The sections along the longitudinal axis of the bone tunnel were cut at 5 μm and stained with hematoxylin and eosin (HE) and safranin O for histological observation. For immunohistochemical staining, hydrogen peroxide was first used to block endogenous peroxidase. Then, the sections were treated with pepsin for 20 min. Monoclonal antibodies against collagen I (Abcam, England), collagen III (Chemicon), and osteocalcin (Abcam, England) were incubated with the sections at room temperature for 4 h. Then, the sections were incubated with biotinylated goat anti-mouse secondary antibody (Univ, Shanghai, China) at room temperature for 1 h. After streptavidin peroxidase was applied, 3,30-diaminobenzidine was utilized as a chromogenic agent, and hematoxylin was used for background staining. All staining and viewing procedures were performed under the same conditions, and typical sections were selected to assess osteointegration at the tendon-bone interface.

### Micro-CT

**Fig. 3:**
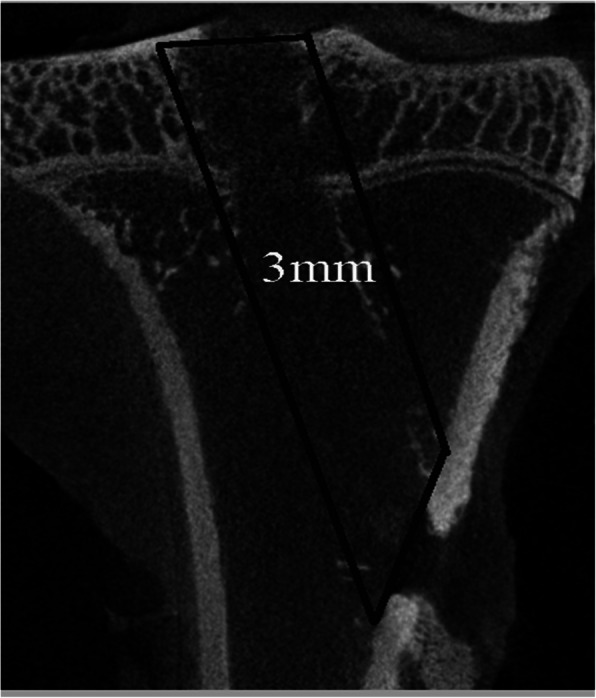
Schematic illustrating that a 3.0-mm-wide cylindrical volume of interest (VOI) was centered along the longitudinal axis of the tibial bone tunnel from the proximal to the distal attachment site

The specimens for micro-CT examination (*n*=6 for each group) were stored at −80°C immediately after harvest. Before the evaluation, the femur-graft-tibia complex was thawed to room temperature. Transverse, coronal, and sagittal images of the tibial bone tunnel were reconstructed by high-resolution micro-CT (36 μm thickness with isotropic resolution, SkyScan 1176, Bruker, Belgium). Therefore, the osteointegration at the tendon-bone interface could be determined by screening all images of each specimen. A 3.0-mm-wide cylindrical volume of interest (VOI) was centered along the longitudinal axis of the tibial bone tunnel from the proximal to the distal attachment site (Fig. 3). The VOI contained the newly formed mineral tissue surrounding the tunnel and the graft because the tunnels were drilled with a 2.0-mm drill bit. All indexes, including the bone volume fraction (BV/TV, %), trabecular number (Tb.N, 1/mm), trabecular separation (Tb.Sp, mm), trabecular thickness (Tb.Th, mm), structure model index (SMI), and bone mineral density (BMD, g/cm^3^), were determined based on the total number of voxels and the number of bone voxels in the VOI.

### Biomechanical testing

**Fig. 4:**
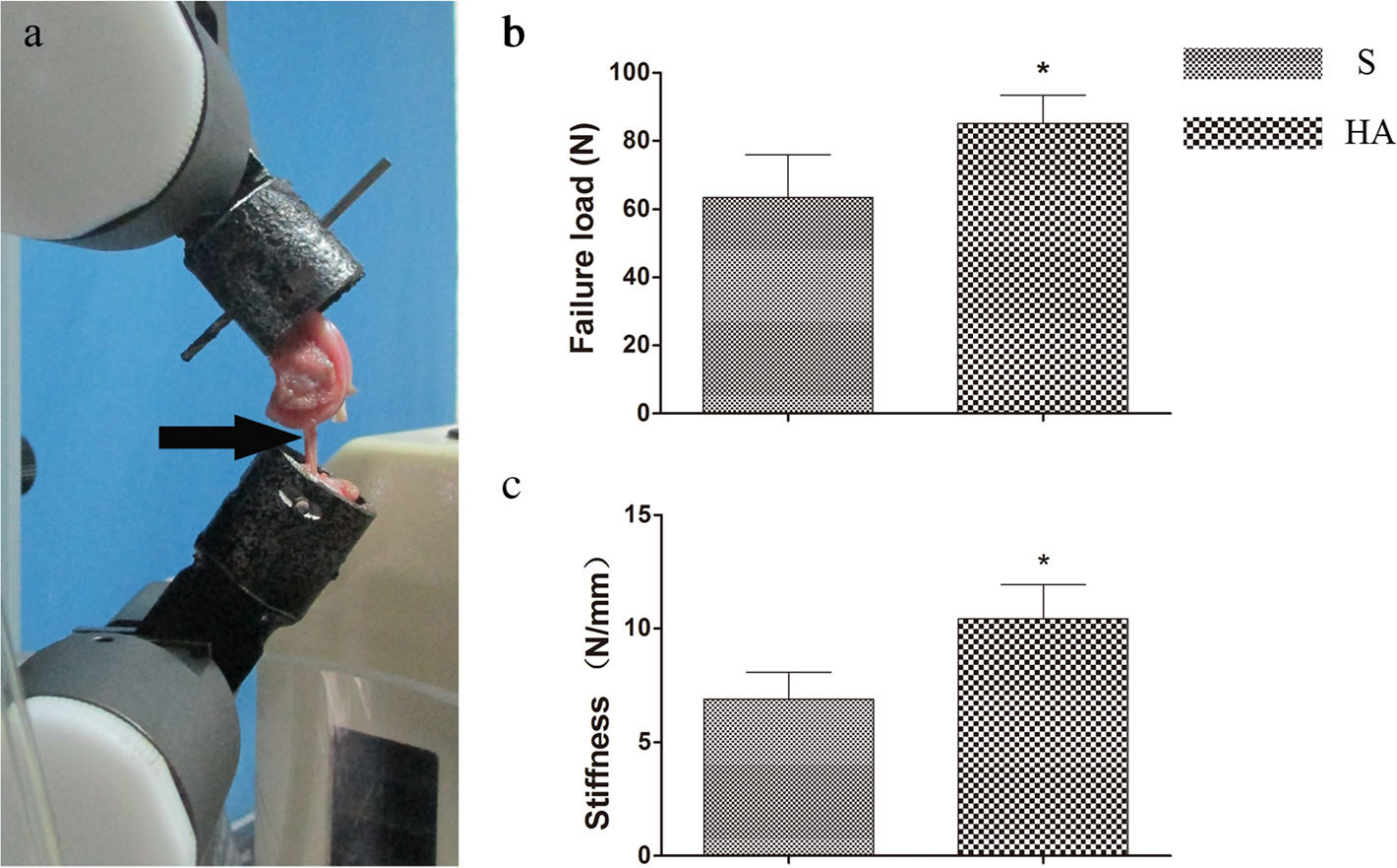
The femur-graft-tibia complex was fixed in custom iron tubes and clamped to an Instron machine for biomechanical testing (**a**, the black arrow points to the graft). The average failure load in the HA group was significantly greater than that in the S group at 16 weeks postoperatively (**b**). A significant difference in stiffness was also detected between the two groups (**c**). Asterisk indicates a significant difference between groups

Specimens were processed for biomechanical testing immediately after micro-CT (*n*=6 for each group). Before testing, all soft tissue except the graft in the knee cavity and sutures at the tunnel exits was carefully removed to eliminate confounding factors. The femur-graft-tibia complex was fixed in custom iron tubes that were clamped to a material testing machine (Instron 553A, USA, Fig. 4a). The preload was set at 1 N, and the tensile load continuously increased at a displacement rate of 20 mm/min. During the test, specimens were always kept moist with normal saline solution. The displacement and tensile load were recorded on the load-deformation curve, and stiffness could be calculated by the slope of the curve. We also recorded the site of graft failure (tibial tunnel, midsubstance, or femoral tunnel).

### Observation of osteoarthritis prevention

X-ray anteroposterior (A-P) photographs of the knee joint of the left hindlimb were captured using a Kodak-FX system at 40 kV for 5 ms. The distance between the tibial plateau and femoral condyles was measured to evaluate the level of osteoarthritis. The cartilage surface of the femoral condyles of the left hindlimb was stained with India ink for macroscopic observation [18]. Then, the specimens were fixed, decalcified, embedded in paraffin blocks, and sectioned at 5 μm. Subsequently, the sections were deparaffinized with xylene, hydrated with decreasing concentrations of alcohol, and then subjected to HE staining and safranin O staining. The modified Mankin score was used to evaluate osteoarthritis in the two groups [19].

### Statistical analyses

All quantitative data are expressed as the mean ± standard deviation (SD), and differences in the results between the two groups were detected by Student’s *t* test. *P* < 0.05 was considered to indicate a significant difference.

## Results

### Gross observation

The native ACL in the knee cavity of the left hindlimb was removed at the attachment site and replaced by a rolled-up silk-collagen scaffold or HA/silk-collagen scaffold with both ends modified by HA. No signs of gross infection were observed in either group; all knee joints of the experimental limbs contained clear serous fluid. The regenerated ligament in the knee cavity resembled the native ACL in both groups at 16 weeks postoperatively. Abundant newly regenerated fibrous tissue filled the space in the scaffold, and silk fibers could be discerned. Additionally, a thin layer of synovium-like tissue was observed on the surface of the regenerated ligament in the knee cavity. The luster and color of the regenerated ligament glossy white throughout, which is similar to that of the native ACL.

### Histological and immunohistochemical staining

**Fig. 5:**
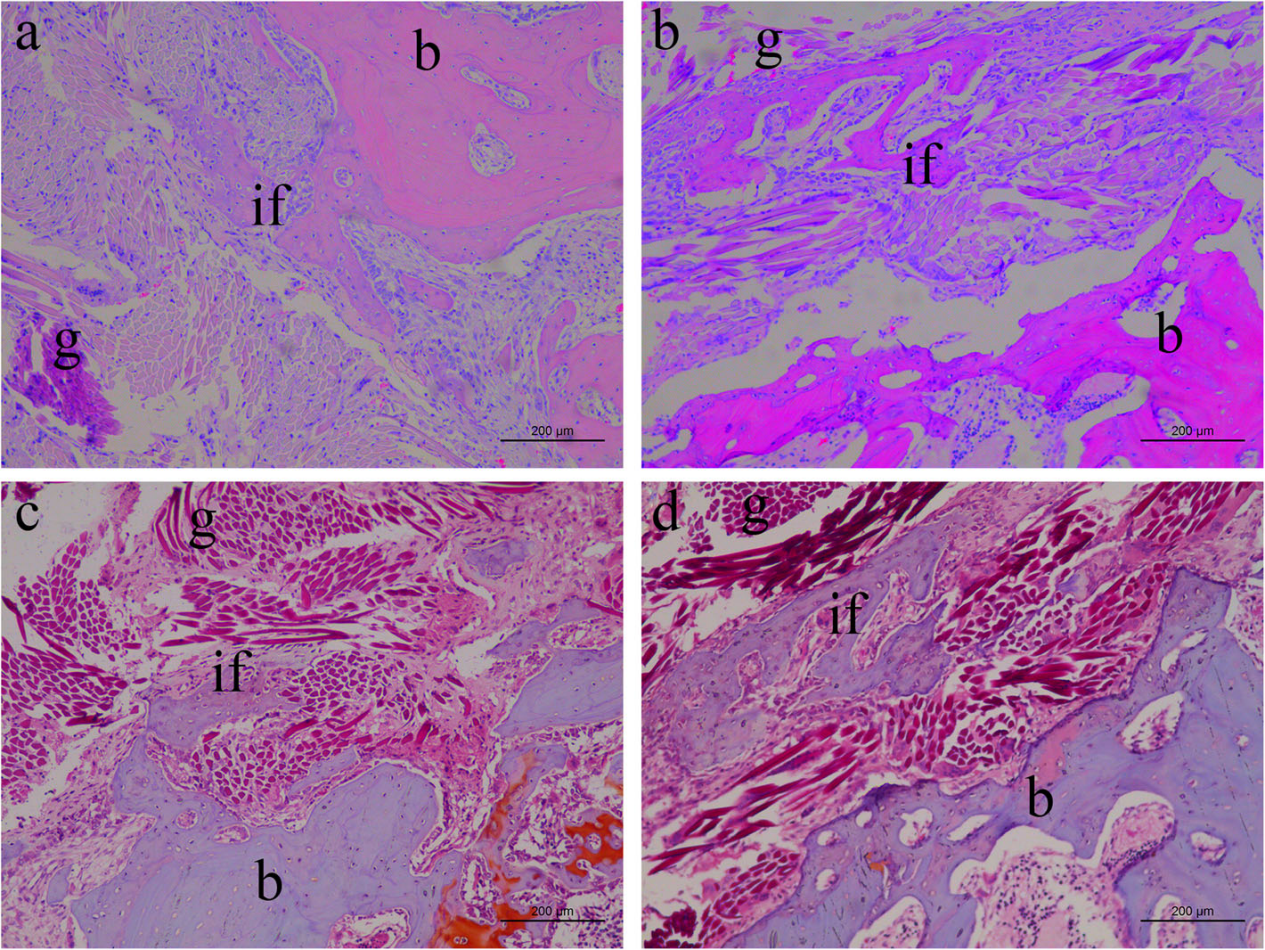
HE staining of the tendon-bone interface. Ingrowth of trabecular bone into the graft was observed in the S group, similar to the findings of our previous study (**a**). Massive trabecular bone formation in the core area of the graft was observed in the HA group (**b**). Safranin O staining revealed more mature osteointegration at the tendon-bone interface in the HA group (**d**) than in the S group (**c**). b, bone; if, interface; g, graft

At 16 weeks postoperatively, much regenerated tissue could be seen in the core and peripheral areas of the grafts in both groups. The grafts showed close contact with the native tissue. In the S group, osteointegration was observed at the tendon-bone interface with bone ingrowth into the graft, as described in our previous study (Fig. 5a) [2]. In the HA group, more mature trabecular bone was observed in the deeper part of the graft, indicating that more osteointegration occurred (Fig. 5b). Moreover, safranin O staining provided clearer images of tissue regeneration and osteointegration. The grafts were surrounded by massive amounts of collagen and newly formed bone. More bone formation was clearly determined in the HA group than in the S group (Fig. 5c and d).

**Fig. 6:**
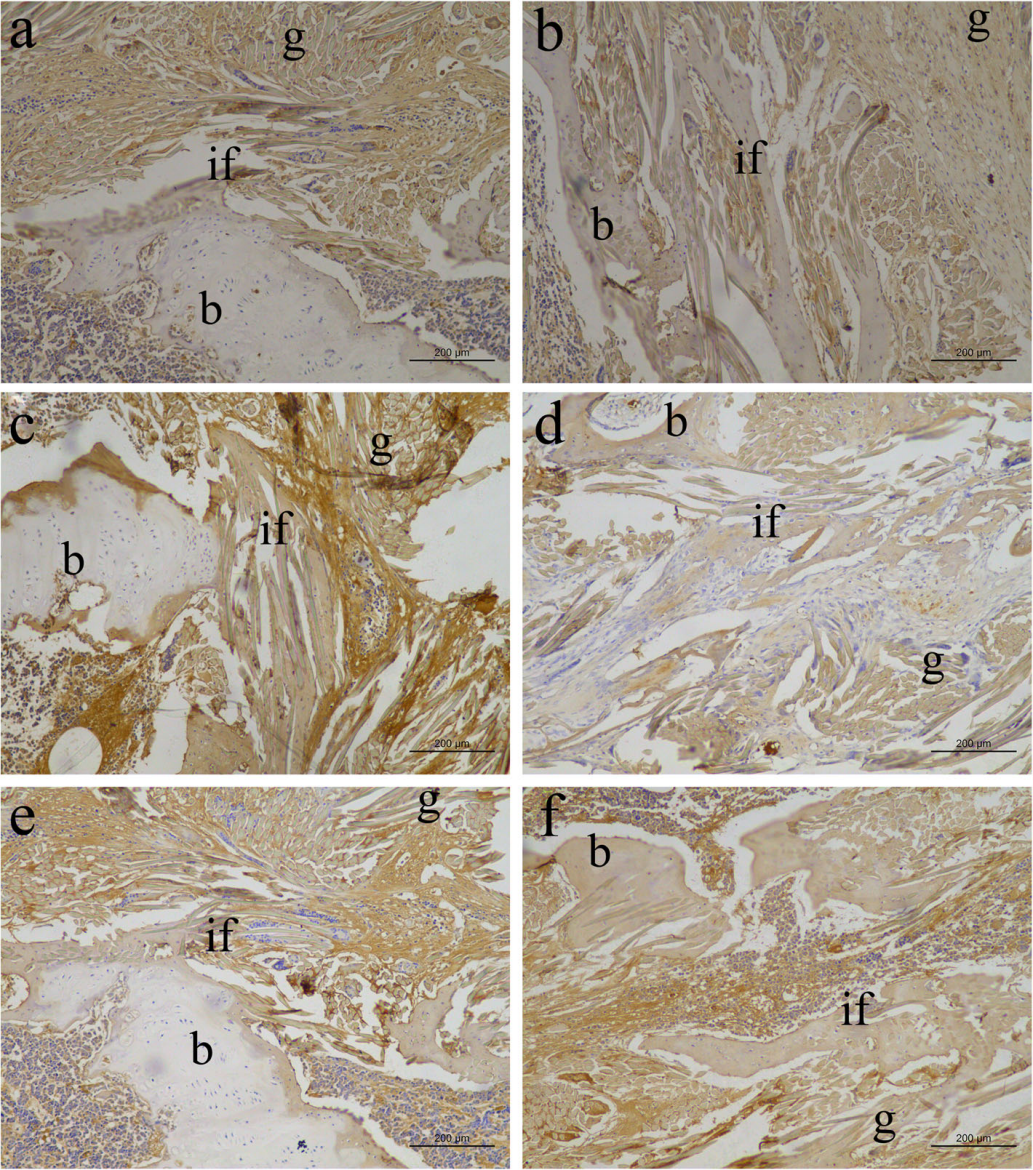
Immunohistochemical staining for collagen I (**a**, **b**), collagen III (**c**, **d**), and osteocalcin (**e**, **f**) in the S group (**a**, **c**, **e**) and HA group (**b**, **d**, **f**). b, bone; if, interface; g, graft

Immunohistochemical staining was used to evaluate extracellular matrix (ECM) production at the tendon-bone interface. More collagen I deposition was observed in the HA group than in the S group, indicating progressive bone matrix regeneration (Fig. 6a and b). On the other hand, collagen III was expressed at a low level in the recovered area in the HA group (Fig. 6c and d). Osteocalcin is abundant in developing bone, and its faded staining in the HA group may implicate major recovery after implantation of the HA/silk-collagen scaffold (Fig. 6e and f).

### Micro-CT

**Fig. 7:**
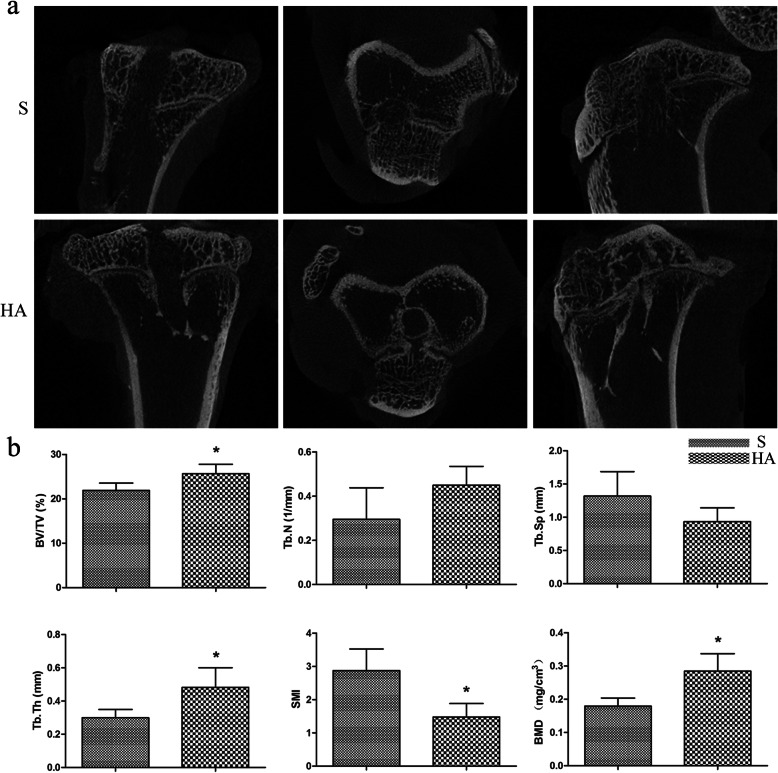
(**a**) Representative transverse, coronal, and sagittal micro-CT images from the two groups. (**b**) Micro-CT evaluations of the bone volume fraction (BV/TV), trabecular number (Tb.N), trabecular separation (Tb.Sp), trabecular thickness (Tb.Th), structure model index (SMI), and bone mineral density (BMD). Asterisk indicates a significant difference between groups

Images of the bone tunnels were reconstructed by high-resolution micro-CT, and the newly regenerated mineralized tissue in the bone tunnels could be easily determined by screening all slices of each sample. At 16 weeks postoperatively, the micro-CT images showed a more obvious signal in the tibial bone tunnel in the HA group than in the S group, indicating more mineralized tissue formation at the tendon-bone interface in the HA group (Fig. 7a). At 16 weeks postoperatively, the BV/TV of the VOI in the HA group was significantly greater than that in the S group (21.91±1.65 for the S group and 25.67±2.10 for the HA group; *p*=0.006). Meanwhile, there were also significant differences detected in the Tb.Th, SMI, and BMD in the HA group compared with the S group (*p*=0.006, 0.01, and 0.001, respectively). No significant difference in the Tb.N or Tb.Sp was detected between the two groups (*p*=0.052 and 0.056, respectively; Fig. 7b).

### Biomechanical testing

In the evaluation of the failure mode, all grafts in both groups failed by partially tearing inside the bone tunnel at 16 weeks postoperatively. The average load to failure in the HA group was significantly greater than that in the S group (HA, 85.07±8.30 vs. S, 63.47±12.65 N; *p*<0.05; Fig. 4b). A significant difference in stiffness was also detected between the two groups (HA, 10.42±1.51 vs. S, 6.89±1.19 N/mm; *p*<0.05; Fig. 4c).

### Prevention of osteoarthritis

**Fig. 8:**
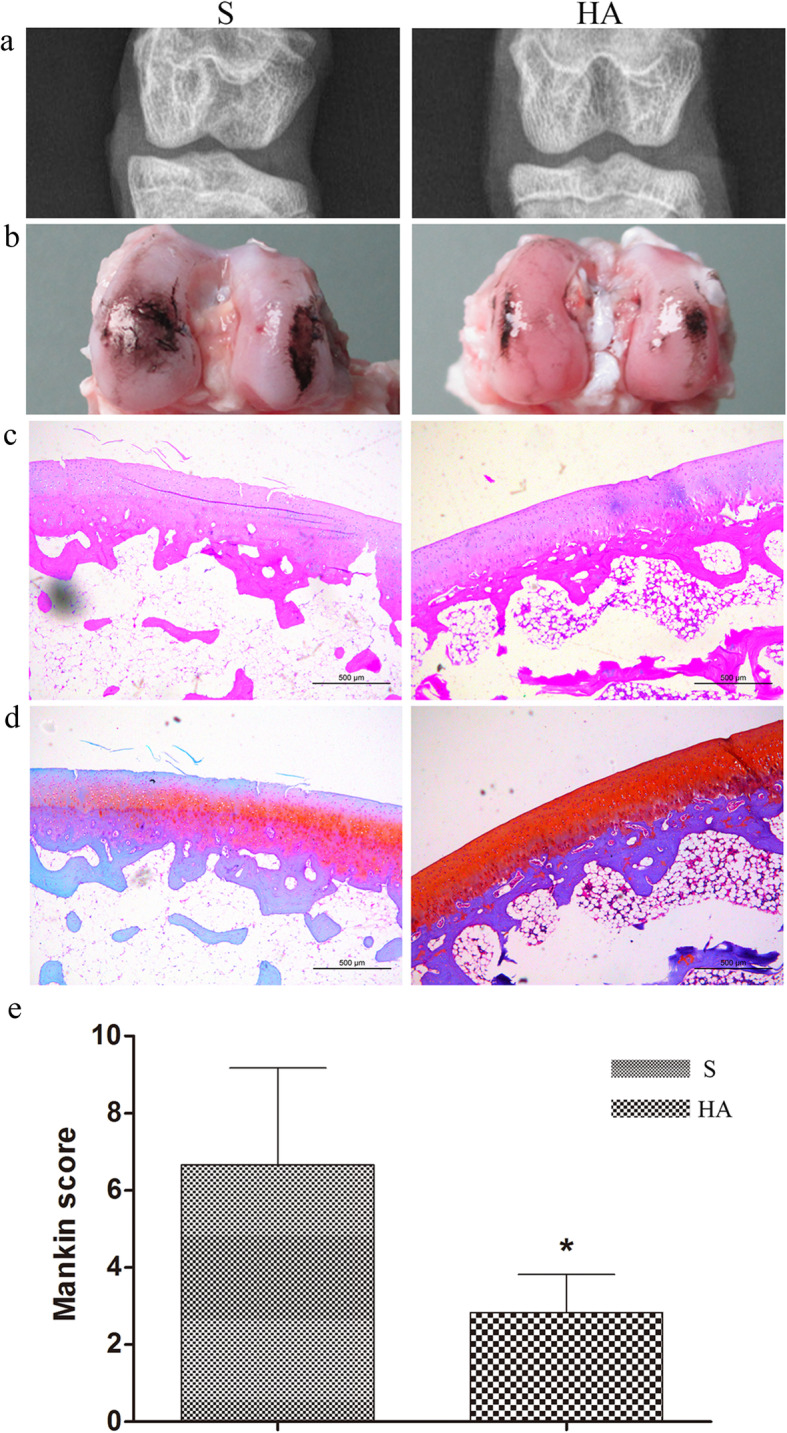
(**a**) Radiological analysis of the knee joint after treatment with the silk-collagen scaffold (S) and HA/silk-collagen scaffold (HA). (**b**) Gross observation of the cartilage surface of the femoral condyles stained with India ink to show the defects. (**c**) HE staining and (**d**) Safranin O staining of the cartilage surface of the femoral condyles. (**e**) Histological evaluation according to the Mankin scoring system. Asterisk indicates a significant difference between groups

Cartilage always degenerates following injuries to the ACL, and an effective therapeutic method should prevent osteoarthritis in addition to regenerating the ACL. Radiological analyses were performed to compare joint knee degeneration between the two groups. Radiographic images demonstrated that joints in the HA group maintained a normal joint space with fewer osteophytes than joints in the S group, indicating that implantation of the HA/silk-collagen scaffold resulted in a more stable joint than that of the silk-collagen scaffold (Fig. 8a). At 16 weeks postoperatively, distinct abrasion of the femoral condyles was observed in the S group, as evidenced by intense staining with India ink (Fig. 8b). The articular surface was less affected in the HA group. Histological images (HE and safranin O staining) showed a regular and smooth articular surface with minimal roughness in the HA group. However, clear fissures and visible fibrillation were present in the S group, which was consistent with the macroscopic results, and weakly stained cartilage of the articular surface was also observed (Fig. 8c and d). The average Mankin score in the HA group was significantly lower than that in the S group, revealing slower degeneration of articular cartilage in the HA group (Fig. 8e).

## Discussion

Applications of cytokines/growth factors and bone marrow mesenchymal stem cells (BMSCs) have become very popular in tissue engineering, especially for spinal fusion and tendon/ligament regeneration [20–23]. However, many problems exist when these technologies are applied in vivo; for example, the final fate of the implanted cells is not clear. However, it has been reported that BMSCs remodel themselves to secrete more extracellular matrix (ECM), which is suitable for ligament regeneration, when they are implanted on silk fibroin scaffolds [24]. Additionally, more ECM deposition for bone formation at the tendon-bone interface is expected after ACL reconstruction [25]. Therefore, the applications of BMSCs and scaffolds need to be modified for better results. Recently, the codelivery and controlled release of cytokines/growth factors with scaffolds has become a trend in tissue engineering, and BMP-2 is an often-used factor for bone regeneration [26–29]. A BMP-2-loaded, bioactive and bioresorbable scaffold fabricated from caprolactone and β-tricalcium phosphate could act as a graft substitute by providing a suitable environment for bone regeneration in a porcine model of interbody spinal fusion [20]. Although bone formation was promoted, several concerns remain, such as the easy loss of cytokine/growth factor bioactivity because of rapid diffusion and microenvironmental changes, dosages being much higher than normal, and the cascade effects continuing to be controversial [30, 31]. Some cytokines/growth factors can cause massive inflammatory reactions when implanted systemically into patients with contraindications [30].

Key components of successful osteointegration at the tendon-bone interface include a biocompatible scaffold fit to the bone interface, progenitor cells, and osteoinductive factors. In our previous study, the silk-collagen scaffold was proven to be a biocompatible scaffold that induced cell infiltration for ligament regeneration and bone ingrowth for tendon-bone healing [2]. Second, host cells around defects that contain BMSCs mainly contribute to initiating and regulating the tendon-bone healing process [32, 33]. HA has been demonstrated to be an effective osteoinductive factor by in vitro and in vivo trials. Therefore, the biomaterial can promote osteointegration at the tendon-bone interface on the material aspect, avoiding the defects of implanted cytokines/growth factors and BMSCs.

Successful ACL reconstruction requires solid tendon-bone healing, but the healing process is slow because the graft in the bone tunnel is separated from the vascular supply and because there is bone loss at the site of injury. During healing, the structure and composition of the native direct tendon-bone interface is not formed, a structurally and biomechanically inferior interface is formed [34]. Previous studies have demonstrated that osteoinductive agents accelerate osteointegration of the tendon graft, with improved tendon-bone healing and mechanical properties [35–37], as does HA [38, 39]. HE and safranin O staining showed that the wounds in the bone tunnel had already recovered in the two groups at 16 weeks postoperatively. The implanted graft and native tissue were tightly connected with each other. New bone tissue grew into the core part of the graft, and a massive amount of trabecular bone was discovered. The results in the S group were similar to our previous findings at 16 weeks postoperatively, and those in the HA group indicated faster bone integration. High cell viability was observed in the two groups, while the scaffold used in the present study did not contain any cells, which further illustrated that implanted cells are not essential for bone integration. Immunohistological staining for collagen I, collagen III, and osteocalcin was performed to further characterize bone integration. Strong collagen I deposition at the tendon-bone interface was observed. The newly formed tissues surrounding the scaffold indicated that the HA/silk-collagen scaffold can promote maturation of the bone matrix. Collagen III is one component of ligament and tendon tissues. Its amount will increase when injured tissues regenerate and remodel and will decrease when the tissues fully regenerate [40, 41]. Therefore, stronger staining was observed in the S group than in the HA group, indicating a slower transition from ligament to bone tissue. Osteocalcin is a marker of terminal osteoblast differentiation influencing bone mineralization [42]. The higher osteocalcin density in the HA group indicated the efficacy of HA/silk in osteogenesis.

The amount of newly formed bone in the tibial tunnels was determined by micro-CT. Micro-CT has been used to evaluate mineralized tissue formation in a rotator cuff model [43], and we used it to determine the amount of bone formed in a tunnel, as in a previous study [9]. Higher BV/TV, Tb.Th and BMD values, which reflect the volume of newly formed bone and thickness of trabecular bone, were detected in the HA group than in the S group. The walls of the bone tunnel in the HA group showed more newly formed bone than those in the S group. The newly formed bone can facilitate direct bonding between the bone and the graft and prevent knee instability associated with bone tunnel enlargement. Bone tunnel enlargement in the knee joint due to bone resorption is a common problem after ACL reconstruction [44]. The new bone formation in the HA group could be more effective in terms of long-term function by preventing joint instability associated with bone tunnel enlargement, according to the present results.

The failure load in the HA group was larger than that in the S group at 16 weeks postoperatively, and a significantly higher stiffness was detected in the HA group. The results of the biomechanical tests were consistent with the histological and radiological findings. When autografts are used to reconstruct the ACL, the tendon-bone healing process comprises a series of cellular events with an orderly transition of graft cell necrosis and host cell ingrowth [33]. The cell types that initiate and regulate tendon-bone healing have not yet been concretely identified [45], and it seems that host cells from the surrounding bone marrow, which contains preosteoblasts, contribute to osteointegration at the tendon-bone interface [32, 33]. In the present study, significant differences in the failure load and stiffness between the HA group and the S group were observed, indicating that the HA on the scaffold exerted an osteoconductive effect on the tendon-bone healing process.

In the present study, osteoarthritis progression following ACL reconstruction was investigated. Osteophyte formation, the joint space width, and the articular surface, which are always disrupted by instability of the knee joint, were assessed by radiological and histological methods. Osteoarthritis was more severe in the S group than in the HA group. Inhibition of the progression of osteoarthritis in the HA group is likely because the HA/silk-collagen scaffold promoted tendon-bone healing, which enhanced joint stability and reduced meniscal injury. Therefore, the results of osteoarthritis occurrence provide comprehensive evidence illustrating that the HA/silk-collagen scaffold can induce better osteointegration at the tendon-bone interface and greater joint stability.

There are some limitations to our study. The animal model we used may not fully mimic human physiological conditions. Additionally, only one time point was selected to evaluate tendon-bone healing, preventing the evaluation of recovery and remodeling throughout the osteointegration process.

## Conclusion

In our present study, an HA/silk-collagen scaffold was fabricated and demonstrated to promote osteointegration at the tendon-bone interface after ACL reconstruction. The HA/silk-collagen scaffold is a promising promoter of osteointegration, as observed in animal trials. Future larger animal and clinical trials are needed.

## Supplementary Information

**Additional file 1: Supplementary data.** Comparison of the micro-CT results and biomechanical test results from the two groups.

## Data Availability

All the data of the manuscript are presented in the paper or additional supporting files.

## Abbreviations

ACL: Anterior cruciate ligament
HA: Hydroxyapatite
SBF: Simulated body fluid
ECM: Extracellular matrix
EDTA: Ethylenediaminetetraacetic acid
HE: Hematoxylin and eosin
VOI: Volume of interest
BV/TV: Bone volume fraction
Tb.N: Trabecular number
Tb.Sp: Trabecular separation
Tb.Th: Trabecular thickness
SMI: Structure model index
BMD: Bone mineral density
SD: Standard deviation
BMSCs: Bone marrow mesenchymal stem cells
BMP: Bone morphogenetic protein
