# Supplementary material for: Anterior cruciate ligament reconstruction in a rabbit model using a silk-collagen scaffold modified by hydroxyapatite at both ends: a histological and biomechanical study
Source: J Orthop Surg Res. 2021 Feb 16;16:139. doi: 10.1186/s13018-021-02281-0 (PMC7885370; doi:10.1186/s13018-021-02281-0)
Supplement: Supplementary file 1 — Additional file 1: Supplementary data. Comparison of the micro-CT results and biomechanical test results from the two groups. [file 13018_2021_2281_MOESM1_ESM.zip › biomechanical test.docx]

| groups | | Failure load（N） | deformation（mm） | stiffness（N/mm） |
| --- | --- | --- | --- | --- |
| HA group | 1 | 85.5 | 9.58 | 8.92 |
|  | 2 | 94.9 | 7.8 | 12.17 |
|  | 3 | 92.7 | 9.7 | 9.56 |
|  | 4 | 86.5 | 7.8 | 11.09 |
|  | 5 | 75.8 | 6.34 | 11.96 |
|  | 6 | 75 | 8.5 | 8.82 |
| Control group |  |  |  |  |
|  | 1 | 49.4 | 6.97 | 7.09 |
|  | 2 | 50.5 | 7.8 | 6.47 |
|  | 3 | 82.2 | 11.9 | 6.91 |
|  | 4 | 70.9 | 10 | 7.09 |
|  | 5 | 67.6 | 13.4 | 5.04 |
|  | 6 | 60.2 | 6.9 | 8.72 |

Results of biomechanical test from the two groups.
