# Supplementary material for: Anterior cruciate ligament reconstruction in a rabbit model using a silk-collagen scaffold modified by hydroxyapatite at both ends: a histological and biomechanical study
Source: J Orthop Surg Res. 2021 Feb 16;16:139. doi: 10.1186/s13018-021-02281-0 (PMC7885370; doi:10.1186/s13018-021-02281-0)
Supplement: Supplementary file 1 — Additional file 1: Supplementary data. Comparison of the micro-CT results and biomechanical test results from the two groups. [file 13018_2021_2281_MOESM1_ESM.zip › Micro-CT results.docx]

**Compare results of Micro-CT**

| groups | Control group | HA group | *P* value |
| --- | --- | --- | --- |
| BV/TV | 21.91±1.65 | 25.67±2.10 | 0.006* |
| BMD | 0.1796±0.0240 | 0.2845±0.0526 | 0.001* |
| Tb.Th | 0.2992±0.0495 | 0.4822±0.1179 | 0.006* |
| Tb.N | 0.2949±0.1426 | 0.4492±0.0856 | 0.052 |
| Tb.Sp | 1.3183±0.3676 | 0.9323±0.2092 | 0.056 |
| SMI | 2.8751±0.6506 | 1.4783±0.4055 | 0.010 |

* indicates significant difference in the two groups.
